# *Vampyrella crystallifera* sp. nov., an Amoeba That Dissolves Entire Algal Cells at a Remarkable Speed

**DOI:** 10.1002/ece3.71089

**Published:** 2025-03-10

**Authors:** Andreas Suthaus, Sebastian Hess

**Affiliations:** ^1^ Division for Biology of Algae and Protozoa, Department of Biology Technical University of Darmstadt Darmstadt Germany

**Keywords:** amoebae, desmids, Endomyxa, peat bogs, predation, streptophyte algae

## Abstract

The vampyrellid amoebae (Order Vampyrellida, Rhizaria) comprise predatory microeukaryotes that inhabit freshwater, marine, and terrestrial habitats. They are known to consume a wide array of prey, which includes microalgae, fungi, and even microscopic animals such as nematodes. Members of the popular genus *Vampyrella* phagocytize the cell contents of filamentous green algae after localized perforation of the cell wall. This feeding strategy, named protoplast extraction, is the hallmark of *Vampyrella* species and vampyrellid amoebae in general. Here, we report on a new species from a German spring fen, *Vampyrella crystallifera* sp. nov., which specifically preys on a unicellular zygnematophyte green alga (*Nucleotaenium* sp.) isolated from the same microhabitat. In contrast to its closest relatives ( *V. lateritia* and *V. pendula* ), *V. crystallifera* does not feed by protoplast extraction but engulfs whole algal cells, followed by the dissolution of the entire prey cell wall. Given the recalcitrant, plant‐like cell walls of the zygnematophytes, this is a remarkable process that might involve enzymes also used by the closely related protoplast feeders. The discovery of *V. crystallifera* again showcases the exceptional diversity of predator–prey interactions found in the Vampyrellida and adds to our knowledge of protist diversity in temperate moorlands.

## Graphical abstract

The new vampyrellid amoeba Vampyrella crystallifera inhabits wet Sphagnum plants in temperate moorlands. Unlike its congeners, it engulfs entire algal cells and breaks them down at a fascinating speed. This represents a feeding habit that was unexpected for the genus Vampyrella and showcases the exceptional diversity of predator–prey interactions found in the order Vampyrellida.

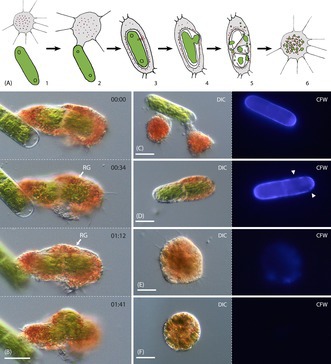

## 1 Introduction

Vampyrellid amoebae are naked, predatory protists belonging to the Rhizaria supergroup (Bass et al. 2009; Hess et al. 2012). They are globally distributed and have been found in a wide range of habitats, including freshwater, moorlands, soil, coastal waters, and the deep sea (Bass et al. 2009; Grell 1985; Lara et al. 2011; Schoenle et al. 2021; Vimercati et al. 2019). Their two‐part life history, including a motile trophozoite stage and the obligatory digestive cyst, is a hallmark of this group (Hess and Suthaus 2022). Otherwise, the vampyrellids exhibit a great diversity of lifestyles and feeding strategies (Hess 2017b, 2017a; Hess et al. 2012; More et al. 2019, 2021; Suthaus and Hess 2023, 2024). The known species consume, for example, green algae, cryptomonads, diatoms, heterotrophic flagellates, ciliates, fungi (including spores), and microscopic animals such as nematodes and rotifers (Bass et al. 2009; Cienkowski 1865; Dobell 1913; Grell 1985; Hess 2017b; More et al. 2021; Valkanov 1970; Weber et al. 1952; Zopf 1885). While some vampyrellids are considered generalist predators (e.g., *Leptophrys vorax*), there are also specialists with narrow prey ranges (e.g., *Vampyrella lateritia* ) (Cienkowski 1865; Hess et al. 2012; Hess 2017a, 2017b; Hülsmann 1983; Zopf 1885). This makes this unique group of amoebae ecologically diverse and versatile.

At present, the order Vampyrellida can be divided into eight phylogenetic subclades at the level of families. While several of these clades grew substantially by the discoveries of the past decade (e.g., the Leptophryidae), the family Vampyrellidae did not. This family branch currently contains only two formal species, *Vampyrella pendula* and *Vampyrella lateritia* , which were already described ~150 years ago and are responsible for the figurative name of the entire vampyrellid order. Both species are specialist predators that extract the cell contents of filamentous green algae after local perforation of the prey's cell wall (Hess et al. 2012). Although they target green algae of different classes, namely zygnematophytes ( *V. lateritia* ) and chlorophytes ( *V. pendula* ), they are relatively similar in morphology and ecology. Both species inhabit algal mats in standing freshwater bodies, exhibit isodiametric, floating trophozoites of bright orange color, and typically attach their digestive cysts to algal prey filaments (Cienkowski 1865; West 1901). None of these species was ever reported to engulf algal cells as a whole or to interact with prey other than filamentous green algae. It is worth mentioning that there are about ~20 other species that were initially assigned to the genus *Vampyrella* (Hess and Suthaus 2022), but that differ substantially from *V. pendula* and *V. lateritia* . Some of them have already been reassigned to other vampyrellid genera and families based on molecular phylogenetic analyses (e.g., *Leptophrys vorax*, *Pseudovampyrella closterii*, *Placopus pedatus*), and several more might follow. To date, these *incertae sedis* vampyrellids cannot be credibly considered as Vampyrellidae sensu Hess et al. (2012).

Here, we report on a new, bright orange vampyrellid amoeba (strain NV.01) isolated from submersed *Sphagnum* plants in a German spring fen. The vampyrellid fed on a yet undescribed desmid (*Nucleotaenium* sp.) by the free capture strategy, involving the phagocytosis of whole algae and the rapid dissolution of the entire algal walls. We examined the trophozoites and cyst stages, performed feeding experiments to assess the prey range specificity of the vampyrellid, and documented the details of its feeding process. Molecular phylogenies based on the gene for the RNA of the small ribosomal subunit (SSU rRNA) suggested that the new vampyrellid originates between the two *bona fide Vampyrella* species, *V. pendula* and *V. lateritia* . Hence, we here introduce *Vampyrella crystallifera* sp. nov., a new member of the Vampyrellidae with unexpected feeding habits.

## 2 Materials and Methods

### 2.1 Natural Samples and Cultivated Organisms

Submersed or wet *Sphagnum* sp. was collected by hand in the spring fen of Neuenhähnen, Waldbröl, Germany (coordinates: 50.840698, 7.535260) and squeezed out, thereby collecting about 30 mL of the run‐off in a polypropylene sampling tube. Samples were transferred into Petri dishes and monitored over several days for vampyrellid cells. Rod‐shaped desmids (Desmidiales, Zygnematophyceae) that appeared morphologically identical to algal cells fed upon by an unknown vampyrellid were isolated with a glass micropipette and transferred to wells of a microtiter plate with the liquid culture medium Waris‐H (McFadden and Melkonian 1986). The isolated algae were kept at 15°C with a 14:10 h day‐night cycle at 10–30 μmol photons m^−2^ s^−1^. One isolate resulted in the monoclonal strain N4, which was tentatively determined as *Nucleotaenium* sp. by morphology. Strain N4 and the additional green algae used in feeding experiments (see results for strain information) were grown in 150 mL Erlenmeyer flasks under the conditions mentioned above and subcultured about every 2 months. The new culture of *Nucleotaenium* sp. (strain N4) was used for the cultivation of the vampyrellid strain NV.01 and is available upon request from the corresponding author.

We obtained another sample from Neuenhähnen and transferred single vampyrellid trophozoites with a glass micropipette to the wells of a microtiter plate with cells of *Nucleotaenium* sp. (strain N4) in half‐strength modified BOG Medium (Eustis 1986), referred to as mBOG/2 in the following. The medium was modified by replacing cyclo acid (cis,cis,cis,cis‐1,2,3,4‐Cyclopentanetetracarboxylic acid) with HEPES buffer (final concentration 0.238 g L^−1^). The resulting vampyrellid strain (NV.01) was maintained in 150 mL Erlenmeyer flasks and T25 polystyrene culture flasks with a rectangular neck and vented cap (Flacon, Corning, NY, USA) at 15°C in the dark. The vampyrellids were fed with *Nucleotaenium* sp. (strain N4) at intervals of about 6 weeks and occasionally transferred to fresh medium. *Vampyrella crystallifera*, strain NV.01, is available from the corresponding author upon request.

### 2.2 Light Microscopy

For the observation and photodocumentation of samples and cultures, including time‐lapse photography, the Motic AE2000 inverted microscope equipped with brightfield and phase contrast optics (Motic Hong Kong Limited, Hong Kong), and with a MikroLive 6.4MP CMOS camera (MikroLive, Oppenau) was used. High‐resolution microscopy with transmitted light was done with the ZEISS Axio Observer 7 inverted microscope equipped with the objective lenses Plan‐Neofluar 20×/0.5, Plan‐Neofluar 40×/1.3, and Plan‐Neofluar 100×/1.3, differential interference contrast optics, and the ZEISS Axiocam 512 color digital camera (ZEISS, Oberkochen, Germany). Epifluorescence microscopy was done with the same microscope by using the Colibri 5 LED illumination system (RGB‐UV) with the 96 HE BFP filter set (excitation 390/40, emission 450/40). To visualize nuclei in strain NV.01, live cells were stained with Hoechst 33528 (Invitrogen, Waltham, Massachusetts, USA) at 1 μg mL^−1^ for 5 min at room temperature and then imaged by fluorescence microscopy. The cell wall degradation in *Nucleotaenium* sp. was observed in cells that were previously stained with 0.02% Calcofluor White (CFW) (Sigma Aldrich, Missouri, USA) in water for 10 min, washed, and then fed to the vampyrellid. The Fiji software (Schindelin et al. 2012) was used for microscopic measurements and Adobe Photoshop CS4 (Adobe Systems, Munich, Germany) for adjusting color balance and contrast of light micrographs.

### 2.3 Feeding Experiments

We selected 21 zygnematophytes and one chlorophyte for feeding experiments with strain NV.01. Once grown to suitable density under the conditions mentioned above 1 mL of the algal cultures was suspended in mBOG/2 medium, distributed to small Petri dishes (diameter 55 mm, VWR Collection), and inoculated with starving trophozoites of the vampyrellid. The experimental cultures were monitored daily for 3 weeks to determine whether the vampyrellid fed, was growing and growth was persistent. Feeding was identified based on the presence of increasing digestive cysts and summarized in two categories, namely “feeding and growth” (+) and “no feeding and no growth” (−). The algal strains used in the feeding experiments are available from the corresponding author or, when listed, from the Central Collection of Algal Cultures (CCAC) at the University of Duisburg‐Essen or the Culture Collection of Algae (SAG) at the University of Göttingen.

### 2.4 DNA‐Amplification, Sequencing, and Sequence Assembly

Vampyrellid DNA was obtained through multiple displacement amplification using the Repli‐G Advanced Single Cell Kit (Qiagen GmbH, Hilden, Germany) according to the manufacturer's instructions. Single trophozoites without food inclusions were isolated with a micropipette, passed through several drops of nuclease free water, and then pipetted into 4 μL of the kit's storage buffer in PCR reaction tubes. The tubes were immediately frozen at −20°C and stored until the DNA amplification procedure. After amplification, the gDNA was diluted 1:100 and used as a template for PCR. The nuclear SSU rDNA gene was amplified by polymerase chain reaction (PCR) using the DreamTaq DNA Polymerase (Fermentas, St. Leon‐Rot, Germany) according to the manufacturer's instructions. The PCR was done with the universal eukaryotic primers EukA and EukB (Medlin et al. 1988) and the following protocol: initial denaturation (95°C for 180″), followed by 30 cycles including denaturation (95°C for 45″), annealing (55°C for 60″), and elongation (72°C for 180″), and a final elongation step (72°C for 300″). PCR products that showed single clear bands by gel electrophoresis were purified using a NucleoSpin Gel and PCR Clean‐up kit (Macherey‐Nagel, Düren, Germany) and subjected to commercial Sanger sequencing by Eurofins Genomics (Eurofins Scientific, Luxembourg). The sequence reads were proofread and assembled using AlignIR (LI‐COR Biosciences, Nebraska, United States).

### 2.5 Molecular Phylogenetics

Preliminary BLAST searches with the SSU rRNA gene sequence of strain NV.01 against the nr/nt database of GenBank (https://blast.ncbi.nlm.nih.gov/Blast.cgi) returned hits with high genetic identity from vampyrellid amoebae (e.g., *Vampyrella lateritia* OK381872, 90.42%). The sequence of interest was added to the most recent multiple sequence alignment of the order Vampyrellida (Suthaus and Hess 2024) and aligned using MUSCLE in SeaView 5.0.4 (Gouy et al. 2021) followed by manual curation. Well‐aligned sites were extracted for further analysis, resulting in a dataset of 93 sequences and 1504 sites, which was then subjected to phylogenetic inference by maximum likelihood (ML) and Bayesian inference (BI). ML trees were inferred with raxmlGUI 2.0.5 using the model GTR + Γ + I, 100 runs, and 1000 bootstrap replicates to assess statistical support. BI was done with Beast 2.7.3 (Bouckaert et al. 2014) using the model GTR + Γ + I, 5,000,000 generations (trees sampled every 1000 generations) and a 25% burn‐in (1,250,000 generations discarded). Stationarity of the BI was confirmed using Tracer 1.7.2 (Rambaut et al. 2018). The SSU rRNA gene sequence of *Vampyrella crystallifera*, strain NV.01, generated in this study was deposited at GenBank under the accession PQ591821.

### 2.6 Generation of Type Material

Strain NV.01 was grown in small Petri dishes (diameter 55 mm, VWR Collection) and then fixed in the dish with 2.5% glutaraldehyde in MT buffer (30 mM HEPES, 15 mM KCl, 5 mM MgSO_4_, 5 mM EGTA, 100 μM DTT, pH 7.0 with KOH) for 20 min at room temperature. Glutaraldehyde was removed by washing with water, followed by post‐fixation with 0.04% osmium tetroxide in water for 20 min at room temperature. The cells were subsequently washed twice with water and removed from the Petri dish bottom by using a cell scraper. The suspended cells were applied to a poly‐L‐lysine‐coated coverslip and allowed to sediment for 30 min, followed by centrifugation at 1000 *g* for 10 min with slow acceleration and deceleration. The cell‐bearing coverslip was passed through a series of ethanol percentages (20%, 40%, 60%, 80%, 90%, and 100%) for 5 min, then dipped into 100% isopropanol for 20 min, and finally mounted on a microscopy slide with a droplet of a Euparal:isopropanol mixture (1:1). The Euparal resin (Carl Roth, Karlsruhe, Germany) was allowed to harden for 24 h before examination.

## 3 Results

### 3.1 Natural Observations and Cultivation

**FIGURE 1:**
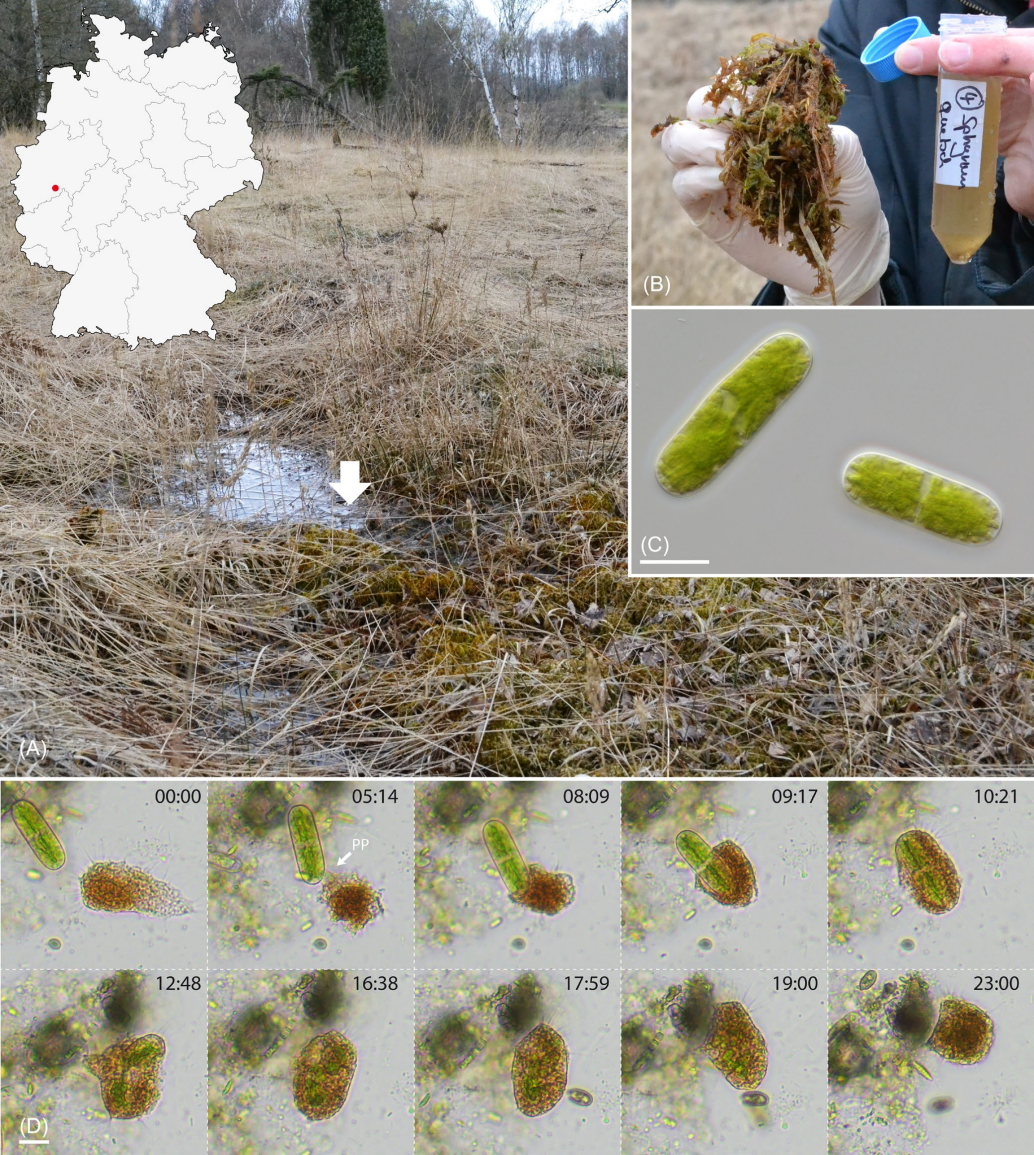
Natural habitat of *Vampyrella crystallifera* (strain NV.01) and first observations on the feeding process; DIC (C), brightfield (D). (A) Spring fen in Neuenhähnen with wet *Sphagnum* (arrow). The location (red dot) is shown on a map of Germany. (B) Representative *Sphagnum* material used for squeeze samples. (C) *Nucleotaenium* sp. (strain N4) colonizing *Sphagnum* plants. (D) Time series (mm:ss) capturing the feeding act of the new vampyrellid in a natural sample. Scale bars: 20 μm (C), 20 μm (D).

In natural samples from the spring fen of Neuenhähnen, Waldbröl, Germany (Figure 1A,B) we observed large, orange cysts of an unknown vampyrellid colonizing wet and submerged *Sphagnum* plants. Additionally, the *Sphagnum* leaflets were studded with numerous cells of a rod‐shaped saccoderm desmid (Figure 1C), here tentatively assigned to the genus *Nucleotaenium* (Desmidiales, Zygnematophyceae). As we did not find any signs of the vampyrellid's feeding habits (e.g., perforated algae), we monitored the natural samples with time‐lapse microscopy and determined *Nucleotaenium* sp. as the vampyrellid's natural prey (Figure 1D, Video 1). The algae were phagocytized entirely within 4 min and subsequently broken down, leaving no cell wall remnants behind. We isolated the desmid, established a laboratory culture (strain N4), and then returned to the habitat to obtain new samples. Several cultivation attempts of the unknown vampyrellid with the cultivated alga in liquid culturing media suitable for other vampyrellids (dilutions of Waris‐H, demineralized water) failed. However, by using a modification of the acidic (pH 5.1), nutrient‐poor BOG medium (Eustis 1986), we finally managed to establish a culture of the unknown vampyrellid (strain NV.01) enabling an in‐depth characterization of *Vampyrella crystallifera* sp. nov.

**VIDEO 1:** Time‐lapse observation of the unknown vampyrellid (resulting in laboratory strain NV.01) in a natural sample from the Neuenhähnen moorland; brightfield, 60× natural speed. Video content can be viewed at https://onlinelibrary.wiley.com/doi/10.1002/ece3.71089

### 3.2 Trophozoite Morphology

**FIGURE 2:**
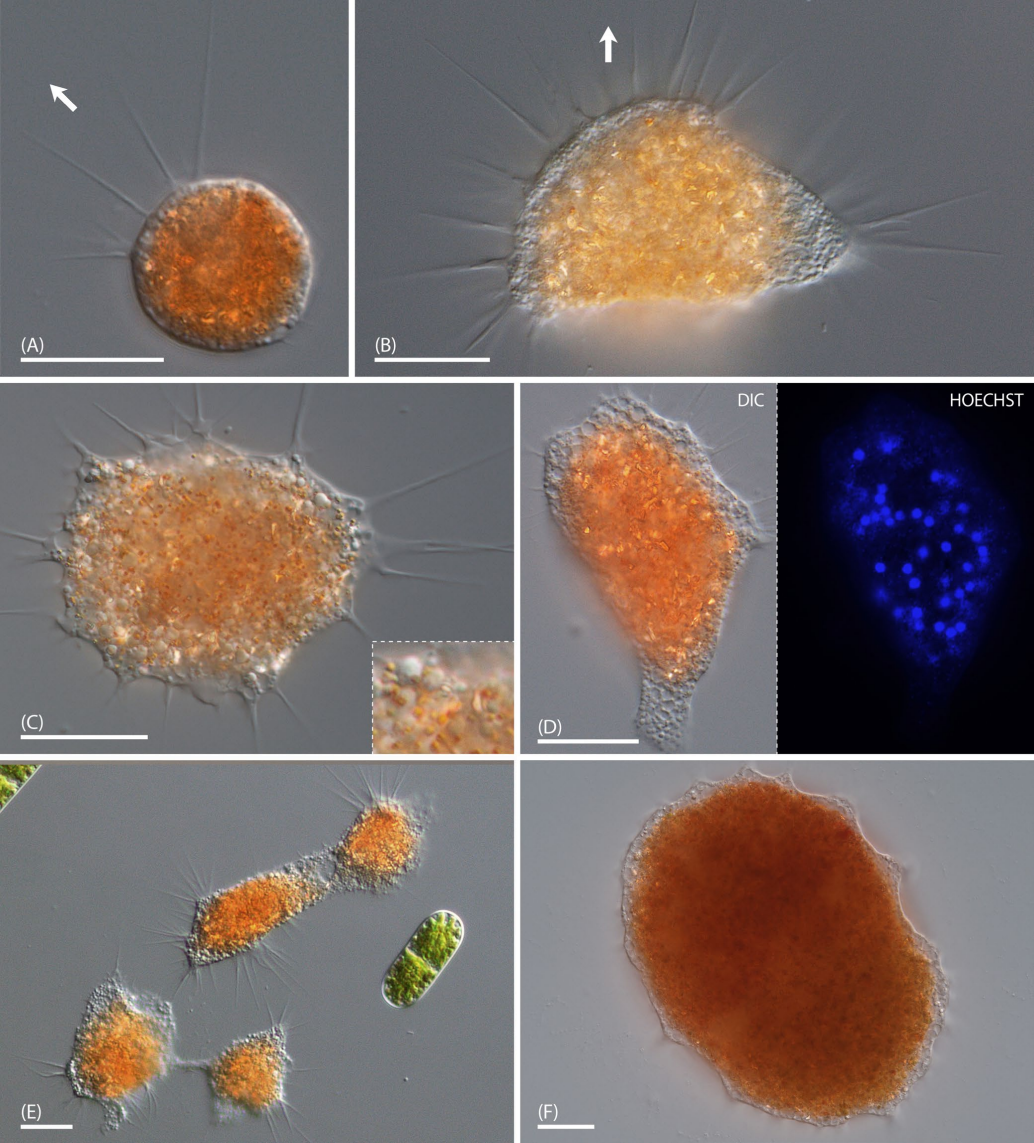
Trophozoites of *Vampyrella crystallifera* (strain NV.01); DIC (A‐F), fluorescence (D). (A) Trophozoite with spherical cell body. The arrow indicates the direction of locomotion. (B) Fan‐shaped trophozoite with anterior filopodia. The arrow indicates the direction of locomotion. (C) Trophozoite with red endoplasmic granules and crystal‐like objects, inset shows magnified details. (D) Hoechst‐stained trophozoite with multiple nuclei and potential bacteria (weak signal) shown in DIC and fluorescence. (E) Trophozoites in fusion. (F) Large plasmodium with condensed cytoplasm. Scale bars: 20 μm (A‐E), 50 μm (F).

Most trophozoites of strain NV.01 exhibited compact cell bodies, with slight amoeboid deformations as they crawled along the substrate (Figure 2A,B). They also could resemble the fan‐like state observed in other vampyrellids of the expanded morphotype (e.g., *Leptophrys vorax*), but did usually not show cytoplasmic tails. Cells floating in the water column were isodiametric. The cell bodies ranged from 20 to 50 μm in size and exhibited relatively straight, tapering filopodia of up to 30 μm in length. These filopodia lacked any visible granules and, in some instances, showed basal branching (Figure 2A–C). The trophozoites typically possessed a clear differentiation between a colorless, granular ectoplasm and an orange endoplasm (Figure 2B). The endoplasm appeared very dense and contained minute, red granules and birefringent structures of irregular shape and size (Figure 2B,C). The latter might be crystals of some sort, which are otherwise rare in vampyrellids. The nuclei were difficult to discern by transmitted light microscopy but could be visualized by fluorescence microscopy after staining with Hoechst dye. The cells of strain NV.01 contained dozens of nuclei measuring about 2 μm in diameter and small fluorescent structures, which might represent intracellular bacteria (Figure 2D). The trophozoites readily fused and formed larger amoebae. In those instances, the colorless ectoplasm fused well before the orange endoplasm (Figure 2E). In cultures depleted of food we observed the formation of plasmodia exceeding 350 μm (Figure 2F), similar to many other vampyrellids.

### 3.3 Prey Range Specificity and Feeding Process

**FIGURE 3:**
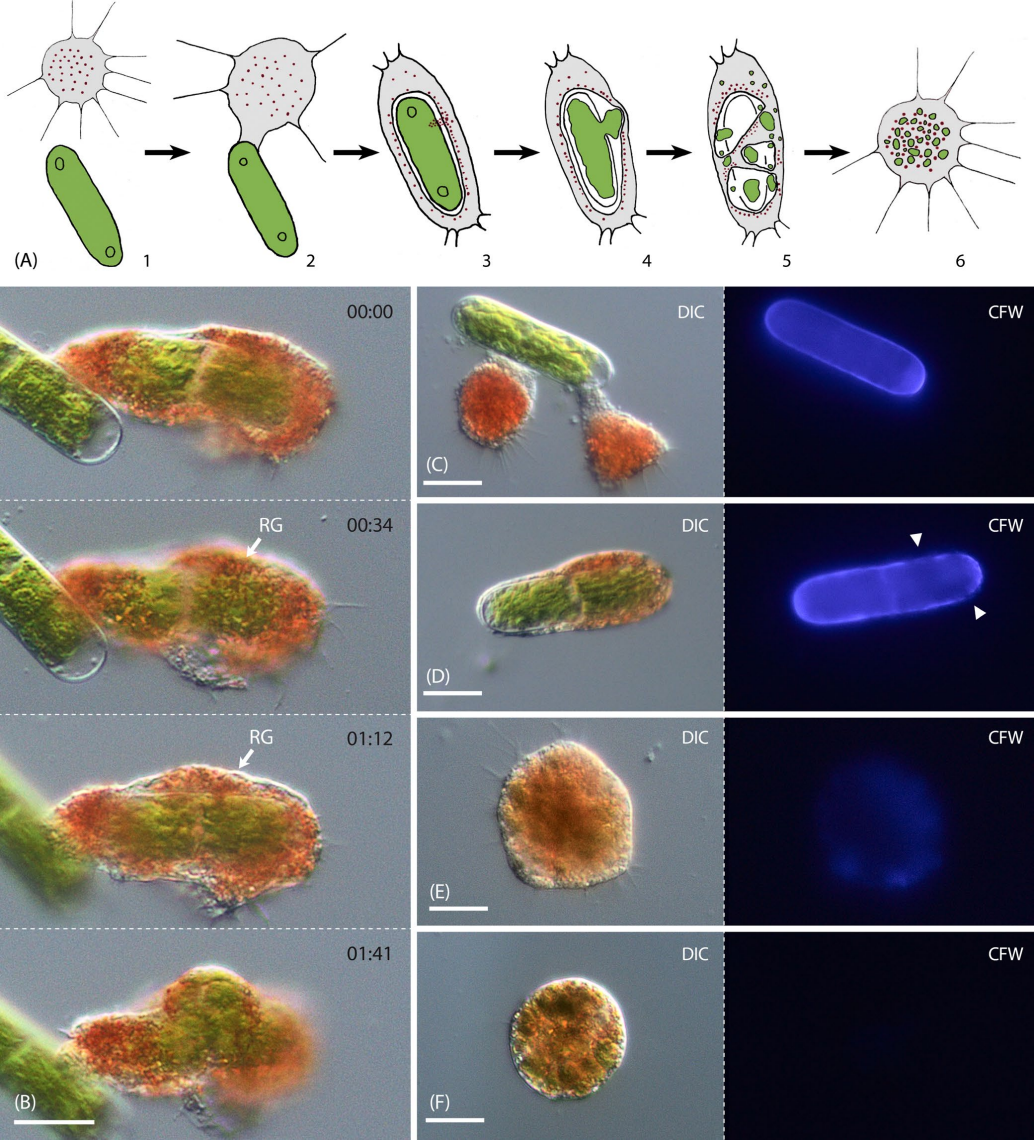
Feeding process of *Vampyrella crystallifera* (strain NV.01); DIC and fluorescence (B‐F). (A) Schematic of the feeding process: (1) the amoeba approaches a *Nucleotaenium* cell; (2) the amoeba attaches to the *Nucleotaenium* cell with a pseudopodium; (3) red granules arrange around the engulfed *Nucleotaenium* cell and accumulate at a specific site; (4) the algal cell wall ruptures at the site of the red granule accumulation; (5) the algal cell wall dissolves and the algal cell contents are packaged into small vacuoles; (6) amoeba after feeding without any algal cell wall material. (B) Time series (mm:ss) of cell wall rupture. Note the red granules (‘RG’) accumulated close to the engulfed alga. (C) Trophozoites attached to a CFW‐stained *Nucleotaenium* cell that shows unaltered cell wall fluorescence. (D) Engulfed alga with irregular cell wall fluorescence (arrowheads) indicating ongoing cell wall dissolution. (E) Trophozoite immediately after feeding on CFW‐stained algae without any distinct fluorescence signal. (F) Cell fed with CFW‐stained algae during encystation. Note the absence of any detectable CFW signal. CFW, Calcofluor White. Scale bars: 20 μm.

We tested twenty saccoderm and placoderm desmids, and two filamentous green algae (*Zygnema pseudogedeanum*, *Oedogonium stellatum*) as potential food for strain NV.01. The feeding experiments revealed that the vampyrellid fed exclusively on *Nucleotaenium* sp. (strain N4) isolated from its natural habitat, while all other strains proved unsuitable (Table 1). In every observed feeding act, the vampyrellid used the ‘free capture’ strategy, as shown in Figures 1D and 3A. At first, the trophozoite extended a broad, hyaline pseudopodium towards the algal cell and attached to the prey cell wall. Then the amoeba retracted the pseudopodium and devoured the entire algal cell. After a few minutes in the vampyrellid cell body, the algal cell ruptured, and its cytoplasm was partially ejected (Figure 3A_4_,B). The algal cell then disintegrated entirely and was divided into smaller units, potentially separate food vacuoles. During this process, the food inclusions changed color from bright green to greenish brown, indicating the onset of digestive processes. The entire feeding act took 20–30 min, and the disintegration of an internalized algal cell took only 10–15 min. To ascertain the fate of the algal cell wall, we fed strain NV.01 with CFW‐stained *Nucleotaenium* cells and documented the cell wall fluorescence over time. The cell wall signal of incorporated algae decreased during food processing, and the progressing loss of clear signal at defined areas indicated the dissolution of the algal wall (Figure 3C,D). Trophozoites with brownish food inclusions (well after finishing feeding) only retained a slight, diffuse CFW fluorescence (Figure 3E), which was absent in early digestive cysts (Figure 3F). Hence, the feeding process of strain NV.01 includes phagocytosis of an algal cell, cell wall rupture, dissolution of the entire algal wall, and packaging of algal cytoplasm in small food vacuoles, which then undergo digestion (Figure 3A). Interestingly, we observed the accumulation of red, endoplasmic granules along the algal cell wall, especially at the site of the initial cell rupture (‘RG’ in Figure 3B, Video 2).

**TABLE 1:** Results of the feeding experiment with strain NV.01 and selected green algae.

Algal species	Algal strain	Feeding
*Actinotaenium silvae‐nigrae*	CCAC 0140	−
*Closterium* cf. *intermedium*	C250_SH	−
*Closterium cornu*	CCAC 1125	−
*Closterium gracile*	Cgrac01_SH	−
*Closterium limneticum*	CCAC 2687	−
*Closterium* sp.	C120_SH	−
*Cylindrocystis brebissonii*	SAG 615‐1	−
*Cylindrocystis brebissonii*	CCAC 0118	−
*Cylindrocystis brebissonii*	CCAC 0211	−
*Cylindrocystis* sp.	Cyl‐N1	−
*Euastrum humerosum*	N022	−
*Euastrum oblongum*	N058	−
*Micrasterias americana*	C514	−
*Micrasterias rotata*	D465	−
*Netrium digitus*	Ndigi_01_AS	−
*Nucleotaenium* sp.	N4	+
*Oedogonium stellatum*	CCAC 2231 B	−
*Penium margaritaceum*	CCAC 0215	−
*Pleurotaenium trabecula*	N059	−
*Serritaenia* sp.	OBE.1	−
*Serritaenia* sp.	OBE.sm1	−
*Zygnema pseudogedeanum*	CCAC 0199	−

*Note:* The behavior of NV.01 was categorized in “feeding and growth” (+) and “no feeding and no growth” (−). Some microalgae were identified by morphology, sometimes tentatively.

Abbreviations: CCAC, Central Collection of Algal Cultures at the University of Duisburg‐Essen; SAG, Culture Collection of Algae at the University of Göttingen.

**VIDEO 2:** Co‐localization of red granules during cell wall dissolution of strain NV.01, brightfield, 25× natural speed. Video content can be viewed at https://onlinelibrary.wiley.com/doi/10.1002/ece3.71089

### 3.4 Digestive and Resting Cysts

**FIGURE 4:**
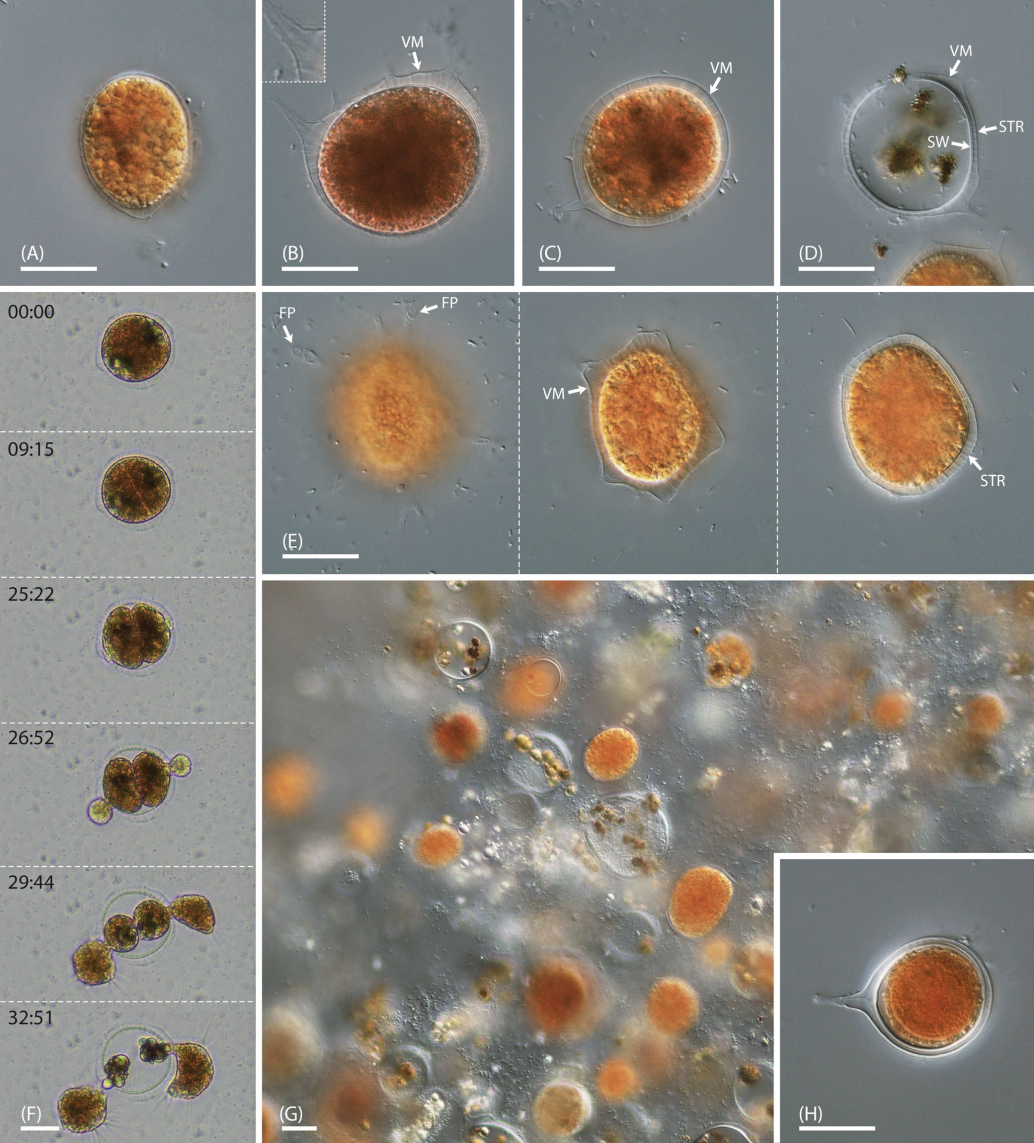
Cyst stages of *Vampyrella crystallifera* (strain NV.01); DIC (A–E, G, H) and brightfield (F). (A) Early‐stage digestive cyst with greenish food inclusions. (B, C) Late‐stage digestive cysts with orange coloration and brown food remains. Note the pronounced velum with strands and protuberances (inset). (D) Empty cyst with brown food remnants and two distinct cell walls. (E) Focal series through a late‐stage digestive cyst revealing the foot‐like protrusions of the velum attaching the cyst to the substrate. Note the fine strands connecting the velum and inner cyst wall. (F) Time series (mm:ss) of hatching amoebae after internal plasmotomy. (G) Biofilm of a late‐stage culture with numerous digestive cysts. (H) Resting cyst with internal spore and two distinct cell walls. FP, foot‐like protrusion; STR, strands; SW, secondary cyst wall; VM, velum. Scale bars: 20 μm.

After the uptake of one to three *Nucleotaenium* cells, the trophozoites of strain NV.01 entered the digestive cyst stage. The digestive cysts had round or nearly elliptic outlines and measured 20–50 μm in their longest dimension. They were green‐brown shortly after encystation (Figure 4A), but turned orange over time (Figure 4B,C). The brown food remnants resided in decentralized vacuoles (Figure 4C). The cysts of strain NV.01 exhibited a smooth or slightly spiny, clearly visible velum (‘VM’; Figure 4B–E), which represents the primary wall. Empty cysts also revealed the relatively thick inner (secondary) cyst wall (‘SW’; Figure 4D), which was connected by conspicuous strands (‘STR’) to the velum (Figure 4D,E). Additionally, the cysts of strain NV.01 possessed several foot‐like protrusions (‘FP’) of the velum that run to the substrate (Figure 4E). Each of these protrusions contained a single connecting strand running through its center (Figure 4B, inset). Time‐lapse microscopy revealed internal plasmotomy in strain NV.01, that is, the vampyrellid divided in the digestive cyst stage, resulting in one to four daughter cells (Figure 4F). Based on observations of our laboratory cultures, this species showed exceptionally long cyst maturation times of three or more days. The cultures fed to depletion over a period of about 3 months and could grow to high densities, thereby forming a biofilm (Figure 4G). In depleted cultures and in the presence of *Oedogonium stellatum* (during the feeding experiments) we observed the formation of resting cysts, which exhibited an orange, spherical spore inside two cyst walls (Figure 4H).

### 3.5 Molecular Phylogeny

**FIGURE 5:**
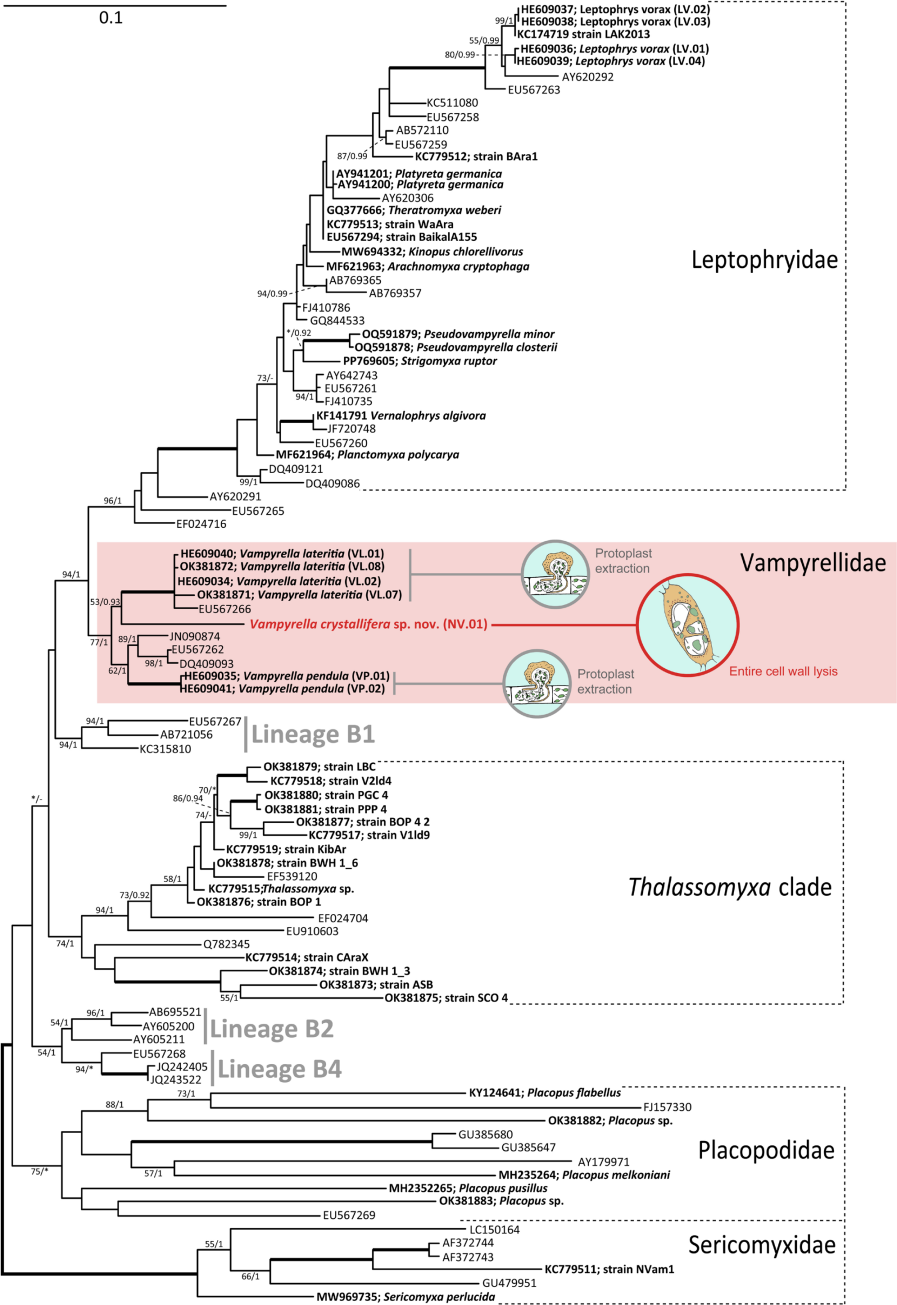
Phylogenetic position of *Vampyrella crystallifera* (strain NV.01) within the Vampyrellida inferred from SSU rRNA gene sequences (93 sequences, 1504 sites, GTR + Γ + I model). The best ML tree of 100 inferences was rooted with the Sericomyxidae. Statistical support is shown as bootstrap support values (%) and posterior probabilities at the branches (ML/BI), except for branches with full support (bold) or support < 50%/0.9 (*). Hyphens (‐) denote branches that were not present in the tree resulting from BI. Sequence names associated with phenotypic information (e.g., cultures or imaged cells) are in bold. Scale bar: 0.1 expected substitutions per site.

We obtained a near full‐length SSU rRNA gene sequence of strain NV.01, which had a relatively close affinity with sequences of *Vampyrella lateritia* (~90% identity) and *Vampyrella pendula* (~89% identity) as assessed by BLAST. Thus, we added the sequence of strain NV.01 to the most recent multiple sequence alignment of the Vampyrellida (Suthaus and Hess 2024) and inferred phylogenetic trees by two different methods. The best maximum likelihood (ML) tree resolved the major vampyrellid sublineages, but with varying support (Figure 5). Bayesian Inference showed similar results as indicated by the posterior probabilities at the branches in Figure 5. Strain NV.01 was confidently placed in the family Vampyrellidae (bootstrap support 77%, posterior probability 1), which to date conforms to the genus *Vampyrella*. The sequence forms a separate branch between the two characterized species *V. lateritia* and *V. pendula* . It is in a sister position to *V. lateritia* , which, however, is not supported.

## 4 Discussion

Our molecular results revealed that the studied vampyrellid constitutes a phylogenetically distinct strain of the Vampyrellidae, nested between two well‐characterized species of the genus *Vampyrella*. In fact, the genetic deviation in the SSU rRNA gene to *Vampyrella* species is substantial (90.42% identity with *V. lateritia* ) and much larger than, for example, that of different genera in the family Leptophryidae (~5% deviation). Furthermore, strain NV.01 shows a unique set of phenotypic traits, including abundant crystal‐like particles in the cytoplasm, a surface‐bound locomotion, the foot‐like protrusions of the digestive cyst, and a so‐far unrecognized feeding strategy. Many other vampyrellids have an orange to red coloration, potentially derived from carotenoids (Hess and Suthaus 2022), but abundant crystals have not been reported (Hess et al. 2012; Hess 2017a, 2017b). The protrusions of the velum, which attach the digestive cysts to the substrate, are reminiscent of the stalk observed in the digestive cyst of *V. pendula* (Cienkowski 1865; Hess et al. 2012). Both structures contain a central strand and are likely homologous. Strain NV.01 tends to display a surface‐bound, amoeboid locomotion with deformations of the cell body. This is rather common in vampyrellids of the expanded morphotype, while the two *Vampyrella* species have more compact trophozoites and move by paddling or walking on the filopodia (Cienkowski 1865; Hess et al. 2012; Hülsmann 1982, 1985). This difference might relate to the feeding ecology of the species. Both *V. lateritia* and *V. pendula* consume filamentous green algae, which generally occur as floating algal mats in the water column. In contrast, strain NV.01 consumes substrate‐associated algae that can be easily gathered by a crawling locomotion.

The new vampyrellid appears to be a specialist predator, as it only consumed *Nucleotaenium* sp. from its own habitat. Considering the phylogenetic context, this trophic specialization is not an unexpected finding, as the next relatives of strain NV.01 ( *V. pendula* and *V. lateritia* ) exhibit confined prey ranges as well (Cienkowski 1865; Hess et al. 2012; Hülsmann 1982, 1985). Several strains of *V. lateritia* (VL.06 and VL.08–VL.11 from the lab of the corresponding author) are unable to feed and grow on *Nucleotaenium* sp. (unpublished observations, A. Suthaus and S. Hess). Furthermore, the feeding habit of NV.01 is in stark contrast to other *Vampyrella* species and, so far, unique among the entire vampyrellid order. At first sight, it seems that NV.01 performs usual phagocytosis and prey digestion, which can be found in many other amoebae including vampyrellids (Grell 1985; Hess 2017b; Hess et al. 2012; Zopf 1885). Supposedly, this involves the enclosure of prey in a food vacuole and the subsequent start of the digestive phase by the fusion of lysosomes. However, our light microscopy data revealed that the feeding process of strain NV.01 is more complex. The actual digestive phase is preceded by an internal food manipulation, specifically cell wall lysis, which is a necessity to access and process the algal cell contents. First, the engulfed desmid ruptures at a specific site, which is followed by the fast disassembly of the entire algal cell. This results in the secondary food vacuoles, which enter the actual digestive phase. To some extent, this process resembles the prey manipulation recently described for *Strigomyxa ruptor* (Leptophryidae). This leptophryid amoeba ruptures desmids (*Closterium* spp.) internally as well, but does not dissolve the algal walls and, instead, discards them by exocytosis (Suthaus and Hess 2024). Interestingly, both vampyrellid species contain red cytoplasmic granules which accumulate at the algal wall before rupture and, thus, might play a role in cell wall lysis. As suggested decades ago (Lloyd 1926; Old et al. 1985), we expect that the algal walls are opened and dissolved by lytic enzymes. The sheer speed at which the new vampyrellid dissolves entire desmid cell walls is remarkable and inspires to explore its repertoire of carbohydrate‐active enzymes in the future. Overall, the feeding process of strain NV.01 involves a distinct and early phase of cell wall lysis. Like in other algivorous species of the genera *Vampyrella*, *Pseudovampyrella*, *Placopus* and *Strigomyxa*, this can be understood as an adaptation to the though, plant‐like cell walls of the zygnematophytes (Domozych et al. 2014; Hotchkiss et al. 1989). As no other vampyrellids have been described to lyse the entire cell wall of engulfed algae in the trophozoite stage, we introduce a new feeding strategy for vampyrellids, namely “free capture with complete cell wall dissolution.”

Given the unique phenotypic traits and its distinct phylogenetic position in the Vampyrellidae, we are confident in establishing a new species, *Vampyrella crystallifera* sp. nov., for strain NV.01. Despite the large genetic distance to the most closely related vampyrellid species, we are hesitant to consider this anything but a new species of the genus *Vampyrella*. The diversity of the Vampyrellidae still remains enigmatic, and future discoveries may provide greater insight into whether splitting the family into several genera is reasonable.

## 5 Taxonomy

**Vampyrellida** West, 1901

**Vampyrellidae** Zopf, 1885

***Vampyrella*** Cienkowski, 1865

**Type species:** *Vampyrella pendula* Cienkowski 1865

***Vampyrella crystallifera*** sp. nov.

**LSID:** urn:lsid:zoobank.org:act:568E6540‐AF18‐4877‐BD7F‐73802911EC32.

**Etymology:** *crystallus*, *‐i*, m [Latin] = crystal; fero, fers, ferre [Latin] = to bear, to carry. The epitheton refers to the crystal‐like inclusions found in the cytoplasm of the species.

**Description:** Trophozoites spherical, slightly deformed or fan‐shaped. Cell bodies measuring 20–50 μm, with colorless granular ectoplasm and orange endoplasm. Endoplasm with birefringent, crystal‐like particles and red granules. Filopodia hyaline, straight, tapering and basally forking, measuring up to 30 μm in length. Cells multinucleate with spherical nuclei of about 2 μm. *V. crystallifera* feeds on *Nucleotaenium* sp. by phagocytosis followed by dissolution of the entire algal cell wall and multiplies by internal plasmotomy resulting in up to four daughter cells.

**Differential diagnosis:** Differs from all known Vampyrellida by its prey preference (*Nucleotaenium* sp.) and feeding strategy (free capture with complete cell wall dissolution). Differs from *V*. *lateritia* and *V*. *pendula* in the presence of numerous cytoplasmic, crystal‐like structures and the foot‐like protrusions of the digestive cyst.

**FIGURE 6:**
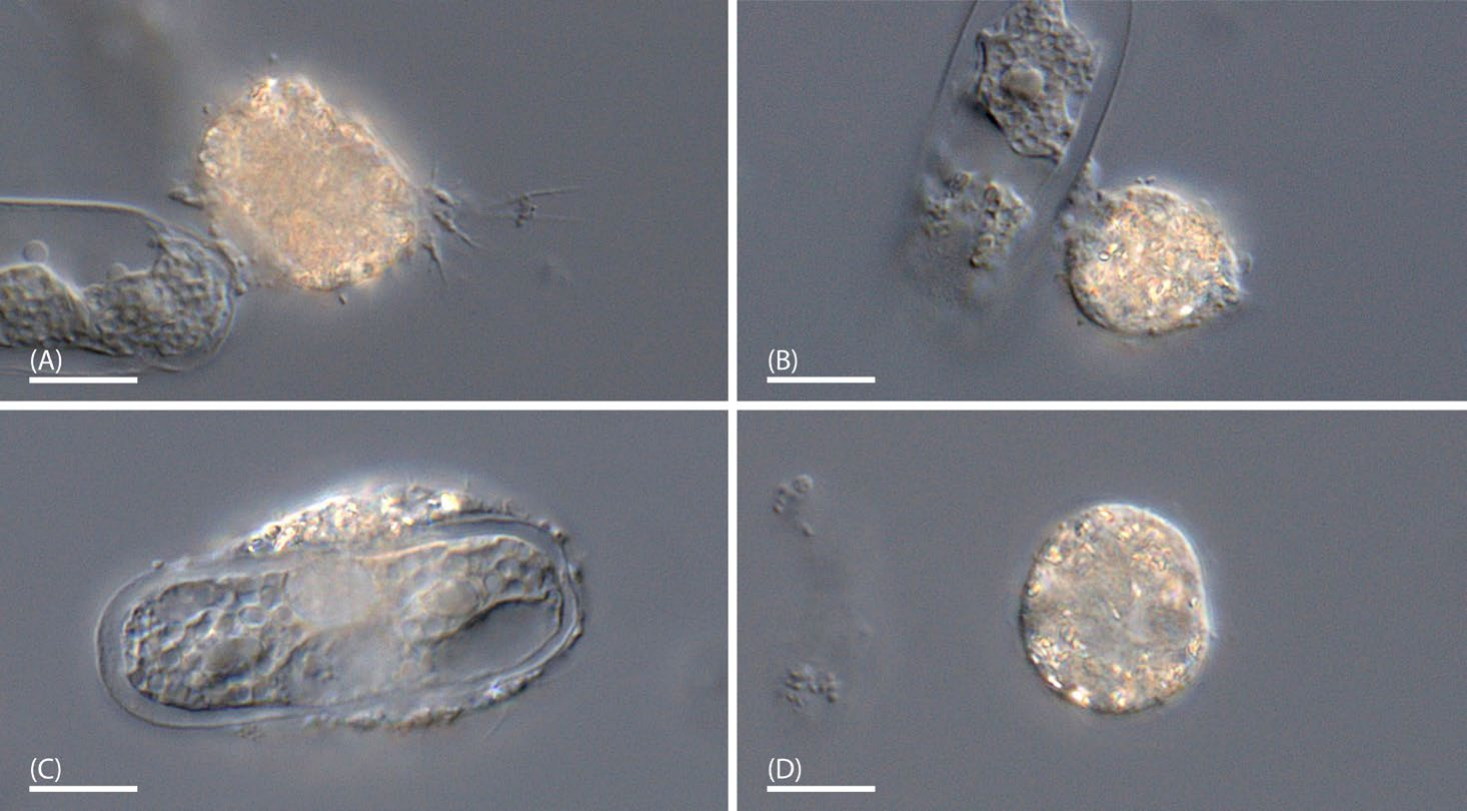
Representative cells of the hapantotype of *Vampyrella crystallifera* (strain NV.01); DIC. (A) Trophozoite with filopodia. (B) Trophozoite presumably attaching to a cell of *Nucleotaenium* sp. (C) Trophozoite with engulfed *Nucleotaenium* cell. (D) Digestive cyst. Scale bars: 20 μm.

**Type material:** A permanent slide (aldehyde/osmium tetroxide fixed cells for DIC microscopy) constituting the name‐bearing hapantotype (article 73.3, ICZN) has been deposited in the “Protists Collection” at the Department of Life Sciences of the Natural History Museum in London (Cromwell Road, London, U.K.) with registration number NHM 2024.11.18.1. Cells of the hapantotype are shown in Figure 6A–D.

**Type generating strain:** NV.01.

**Sequence of type generating strain (SSU rRNA gene):** PQ591821.

**Type habitat and locality:** Squeezed *Sphagnum* moss; Neuenhähnen, North Rhine‐Westphalia, Germany; collected 2022, leg. S. Hess.

## Author Contributions

**Andreas Suthaus:** conceptualization (equal), formal analysis (equal), investigation (lead), visualization (lead), writing – original draft (equal). **Sebastian Hess:** conceptualization (equal), formal analysis (equal), funding acquisition (lead), resources (lead), visualization (supporting), writing – original draft (equal), writing – review and editing (lead).

## Conflicts of Interest

The authors declare no conflicts of interest.

## Data Availability

The genetic data that support the findings of this study have been made publicly available and can be accessed via GenBank (https://www.ncbi.nlm.nih.gov/), accession number PQ591821.
